# Supplementary material for: Machine Learning Ensemble Directed Engineering of Genetically Encoded Fluorescent Calcium Indicators
Source: Res Sq. 2023 Aug 7:rs.3.rs-3146778. Preprint. [Version 1] doi: 10.21203/rs.3.rs-3146778/v1 (PMC10441480; doi:10.21203/rs.3.rs-3146778/v1)
Supplement: Supplement 1 [file NIHPPrs3146778v1-supplement-1.pdf]

Supplementary Files

This is a list of supplementary files associated with this preprint. Click to download.

- SwaitMachineLearningEnsembleDirectedEngineeringofGeneticallyEncodedCalciumIndicatorssupplementaryfiguresandtables.pdf
